# Rare Earth Elements Recovery and Waste Management of Municipal Solid Waste Incineration Ash

**DOI:** 10.1021/acssusresmgt.3c00026

**Published:** 2023-11-29

**Authors:** Yinghao Wen, Lei Hu, Anthony Boxleiter, Dien Li, Yuanzhi Tang

**Affiliations:** † School of Earth and Atmospheric Sciences, Georgia Institute of Technology, 311 Ferst Drive, Atlanta, Georgia 30332, United States; ‡ Department of Geosciences, Georgia State University, 38 Peachtree Center Avenue, Atlanta, Georgia 30303, United States; § Savannah River National Laboratory, Aiken, South Carolina 29808, United States

**Keywords:** rare earth elements, municipal solid waste incineration ash, resource recovery, waste upcycling, sustainable chemistry, waste management

## Abstract

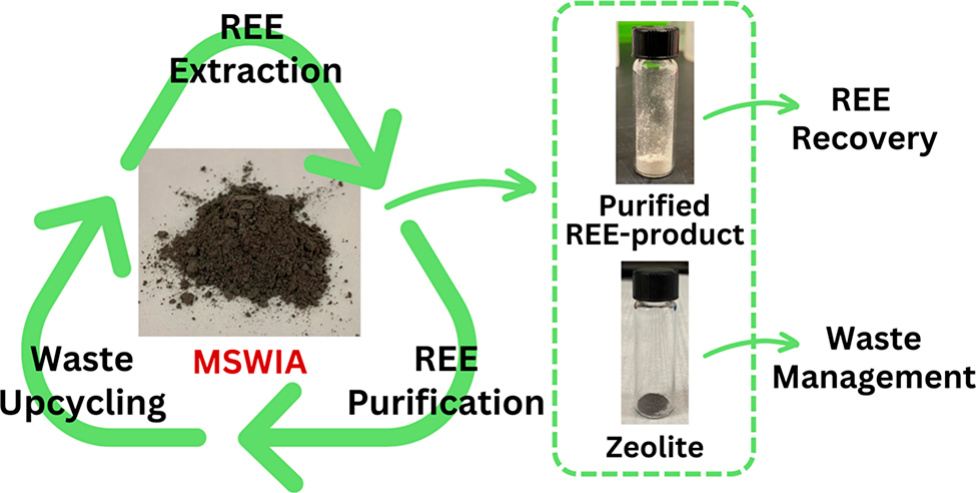

The advancements in high-tech products and pursuit of renewable energy demand a massive and continuously growing supply of rare earth elements (REE). However, REE production from mining is heavily restricted by technoeconomic limitations and global geopolitical tensions. Municipal solid waste incineration ash (MSWIA) has been recently recognized as a potential alternative for REE recovery. This study applies and optimizes a green modular treatment system using organic ligands for effective REE recovery and concentration from MSWIA with minimal generation of secondary wastes. Citrate extracted >80% of total REE at pH 2.0 and ∼60% at pH 4.0. A subsequent oxalate precipitation step selectively concentrated >98% of extracted REE by ∼7–12 times compared to raw MSWIA. Waste byproducts were upcycled to synthesize zeolites, resulting in an overall solid waste volume reduction of ∼80% and heavy metal immobilization efficiency of ∼75% with negligible leaching, bringing the dual benefits of REE recovery and waste management. This work serves as a pioneer study in REE recovery from an emerging source and provides system level insights on the practicality of a simple three-step treatment system. Compared to existing literature, this system features a low chemical/energy input and a light environmental footprint.

## Abstract 2

Rare earth elements are extracted from municipal solid waste incineration ash using a green method, with significant waste residue elimination and heavy metal immobilization.

## 1 Introduction

Rare earth elements (REE) consist of the lanthanide group, yttrium, and scandium. Owing to their unique optical and magnetic properties, REE are indispensable in a wide range of modern high-tech products (e.g., electronics, hybrid/electric vehicles, wind turbines, and photovoltaics) and defense technologies (e.g., missile guidance systems and satellites).^1−5^ Since 1990, global REE production has almost entirely shifted to China, causing the near-complete dependence on REE import and loss of domestic supply chain in many countries.^6−8^ As a result, the U.S and European Union have labeled REE as critical minerals.^9,10^ Although the U.S. has started restoring mine production of REE ores from several domestic sources such as Mountain Pass, California, the U.S. currently still lacks a mature REE manufacturing industry and enough capacity to produce high-grade REE products.^6^ Moreover, mining activities are almost inevitably associated with serious environmental contamination (e.g., toxic gases, extremely acidic wastewater), elevated human health risks, and intensive social resistance.^7,11,12^

Due to these constraints, recovering REE from alternative sources that has been overlooked in the past due to lack of incentives has attracted considerable interest. Compared to mining, recovery of REE from wastes such as industrial residues, end-of-life products, and post-consumer wastes can provide significant benefits and diversify REE resources.^13^ Previous studies have been conducted on the recovery of REE from electronic wastes,^14−16^ coal combustion byproducts,^17−19^ phosphors,^20,21^ mine tailings,^22,23^ and acid mine drainage,^24,25^ and efforts are still ongoing to search for new REE-bearing feedstocks. Municipal solid waste incineration ash (MSWIA), the solid combustion residue of municipal solid waste produced at waste-to-energy facilities, has a massive production in many countries and has been recently recognized as another potentially important REE resource.^26,27^ For land-limited countries such as Denmark, Netherlands, and Germany, reuse of MSWIA in various applications (e.g., road construction and concrete manufacturing) has been strongly encouraged, and the overall utilization rate can reach 60–100%.^28,29^ In sharp contrast, the U.S. is one of the biggest producers of MSWIA (∼8 million tons in 2018), but only less than 5% is beneficially used whereas the rest is landfilled.^30^ The low utilization rate and heavy disposal pose significant management costs and environmental hazards.^31−34^ The total REE content in MSWIA typically ranges from ∼50 to 150 ppm but could be markedly higher if the feedstock contains electronic wastes (e.g., spent magnets) prior to combustion.^26,27^ Thus, the environmental and economic benefits of REE recovery could be significant, considering the huge annual MSWIA production. Development of a feasible technology that recovers REE from MSWIA while relieving the burden of MSWIA management is highly desired.

To date, various technologies have been developed to recover REE from wastes, including hydrometallurgy, pyrometallurgy, solvent extraction, microbial leaching, membrane filtration, and ion exchange.^12,35−38^ Currently, the state-of-art REE manufacturing process relies on hydrometallurgical leaching using concentrated strong mineral acids followed by biphasic solvent extraction.^11^ Despite its effectiveness and high scalability, such a process leaves a heavy environmental footprint and requires intensive chemical/energy input.^35^ Our recent study developed a green three-step treatment system that effectively extracts REE from coal fly ash (CFA).^39^ Thanks to the formation of a soluble metal–ligand complex, using diluted organic acids under a mild pH could achieve comparable REE extraction efficiency as concentrated mineral acids.^40,41^ Unlike many mineral acids, citric acid is non-hazardous and biodegradable, forming a stable REE–citrate complex with a high stability constant (log β ≈ 6.7–11.8).^42−44^ After leaching, separation of REE from other interfering metals (e.g., Mg, Al, and Fe) in the leachate was achieved by adding oxalate. Our PHREEQC calculations predicted that in a Ca-rich solution, oxalate would preferentially react with Ca^2+^ to form calcium oxalate, coprecipitating REE while leaving other metals in the aqueous phase.^39^ These two steps produced not only a purified REE solid product but also waste byproducts at the same time. Hence, in the last step, these waste streams were combined and upcycled to synthesize zeolite. This three-step system features effective REE extraction and purification and reduced secondary wastes. However, in order to further evaluate the overall system practicality, more effort is needed to optimize important parameters in each step (e.g., reaction time, solution pH, chemical dosage), track the fate of heavy metals throughout the system, and assess the feasibility of waste upcycling (characteristics and potential uses of zeolite). In addition, the applicability of this treatment system to other REE-containing feedstocks, such as MSWIA needs to be evaluated.

**Scheme 1:**
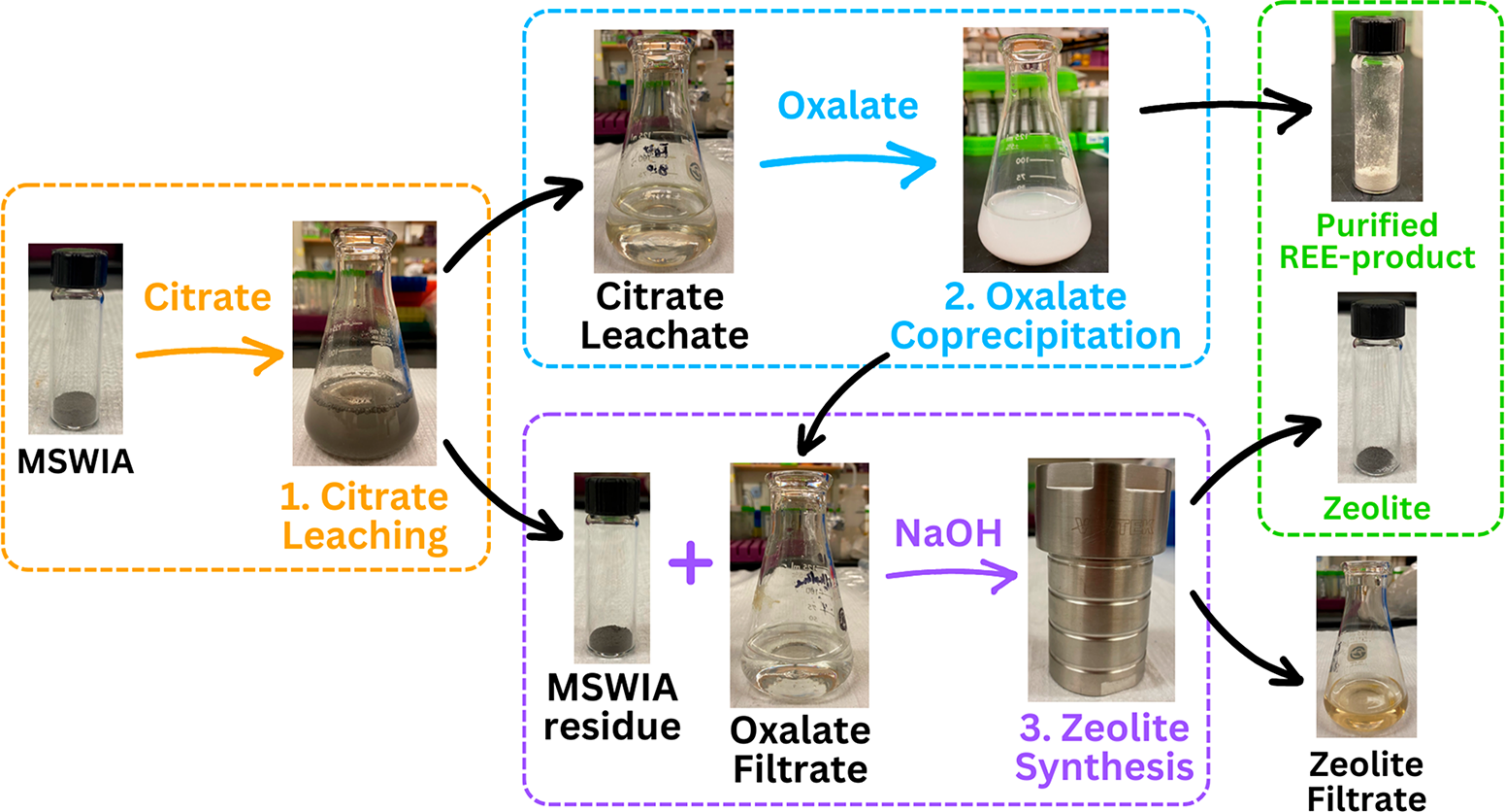
Diagram of the Modular Treatment System in This Study

Although some previous studies have determined the REE concentration in different MSWIA samples,^26,27,34^ very little is known about REE speciation (e.g., chemical species, physical distribution) in MSWIA and the recovery viability (e.g., extraction, purification). Since bottom ash constitutes the majority of MSWIA and typically contains higher REE contents than fly ash,^26^ it was used as the feedstock in this study (hereafter referred to as MSWIA; see Text S2 for more details). The main objectives of this study are (1) testing the performance of the above-mentioned three-step treatment system on REE recovery from MSWIA, (2) optimizing reaction conditions for high REE recovery efficiency and minimal chemical input, and (3) examining the environmental impacts and benefits of the waste upcycling process. Specifically, the optimal leaching conditions of REE from MSWIA were determined, including the MSWIA particle size, reaction time, pH, citrate concentration, and liquid-to-solid ratio. The separation efficiency of REE over non-REE by oxalate coprecipitation at various oxalate dosages and the corresponding enrichment factor were investigated. The zeolite products of waste upcycling were characterized for morphology, crystallinity, and phase. The fate of heavy metals was tracked throughout the system, from coextraction by citrate to immobilization by zeolite products. More importantly, the environmental benefits and impacts of waste upcycling were evaluated by the stability of zeolite products, final wastewater composition, and overall waste volume reduction. This work serves as a systematic investigation and optimization of this three-step treatment system to further examine its overall feasibility in REE recovery from secondary feedstocks. To the best of our knowledge, this is the first systematic study on REE extraction from MSWIA and system level waste management. An overview of the treatment system in this study is illustrated in Scheme 1.

## 2 Materials and Methods

All chemicals used in this study are ACS grade or higher without further purification. Details on the chemicals, materials, ash sample processing, and characterization techniques are provided in the Supporting Information Text S1–S6.

### 2.1 Extraction of REE from MSWIA via Citrate Leaching

A 100 mL portion of 50 mM sodium dihydrogen citrate was prepared in a 200 mL Erlenmeyer flask. The initial solution pH was adjusted to 2.0, 4.0, or 7.0 by using 3 M HCl or NaOH solutions. The total volume of added HCl or NaOH solution was less than 0.5 mL, and thus, their impacts on analyte concentrations were negligible. Desired mass of MSWIA (all three particle sizes) was added to the citrate solution to achieve a liquid-to-solid ratio of 50, 100, 200, or 400 mL/g. The flask was then sealed with parafilm and agitated on an orbital shaker at 240 rpm. To determine the required reaction time to achieve equilibrium, a preliminary kinetic experiment was conducted at pH 4 with a liquid-to-solid ratio of 200 mL/g and reaction time of 24 h. 1 mL of the reaction mixture was extracted using a syringe at various time points. To study the impacts of different pH and liquid-to-solid ratios, leaching experiments were conducted for 4 h. In addition, cycle experiments were also performed to optimize the leaching efficiency (pH 4, liquid-to-solid ratio of 200 mL/g, 8 h) by replenishing 50 mM of citrate at the end of each cycle. The collected samples were filtered through a 0.22 μm cellulose nitrate membrane filter by using vacuum filtration. The filtrate (hereafter termed citrate leachate) was analyzed for elemental composition by ICP-MS, and the solid residue (hereafter citrate residue) was air-dried and stored. The citrate leaching efficiency was calculated by eq 1:
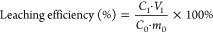
1where *C*_1_ and *C*_0_ (ppm) are the elemental concentrations in citrate leachate and raw MSWIA sample, respectively, *V*_1_ (mL) is the volume of citrate leachate, and *m*_0_ (g) is the mass of ash sample.

### 2.2 Selective Concentration of REE from Non-REE via Oxalate Coprecipitation

To concentrate REE and separate it from non-REE, varied concentrations of sodium oxalate (5, 10, 20, 30, and 40 mM) were added to the citrate leachate. White precipitates were observed to occur immediately. After 30 min, the precipitates and supernatant were separated using vacuum filtration. The filtrate (hereafter oxalate filtrate) was analyzed for elemental composition by ICP-MS, and the solid precipitate (hereafter oxalate precipitate) was dried at 50 °C in an oven overnight and analyzed using XRD. The coprecipitation efficiency of REE or other metals was calculated by eq 2:
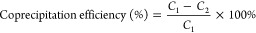
2where *C*_2_ (ppm) is the elemental concentrations in the oxalate filtrate.

The enrichment factor of REE in the oxalate precipitate compared to raw MSWIA sample was calculated by eq 3:
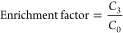
3where *C*_3_ (ppm) is the REE concentration in oxalate precipitate.

### 2.3 Waste Upcycling and Heavy Metal Immobilization via Zeolite Synthesis

Solid and liquid wastes from the upstream extraction and separation steps under optimal conditions (citrate leaching: 50 mM citrate, liquid-to-solid ratio 200 mL/g, pH 4.0, 4 h; oxalate coprecipitation: 10 mM oxalate, 30 min) were combined to synthesize zeolite via a low-temperature hydrothermal method. The citrate residue (from the citrate extraction step) and oxalate supernatant (from the oxalate coprecipitation step) were mixed with 0.5 or 5 M NaOH in a 20 mL Parr hydrothermal reactor at a liquid-to-solid ratio of 50 mL/g. The reactor was heated in an oven at 150 °C for 12 h. At the end of the reaction, the solid products were filtered using vacuum filtration, washed with DI water, dried at 50 °C overnight, and characterized using XRD and SEM coupled with energy dispersive X-ray spectroscopy (EDX). Elemental composition of the liquid filtrate (hereafter zeolite filtrate) was analyzed by ICP-MS. From the initial MSWIA to the final zeolite solids, the overall volume reduction of the system was calculated using eq 4:
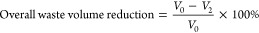
4where *V*_0_ and *V*_2_ are the volumes of raw MSWIA sample and synthesized zeolite, respectively, calculated based on their density and mass. The density of raw MSWIA and zeolites was estimated to be 0.75 and 0.42 g/cm^3^, respectively.

The immobilization of heavy metals in the final zeolite products was calculated by eq 5:
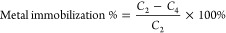
5where *C*_4_ (ppm) is the metal concentration in zeolite filtrate.

The leachability of the zeolite-immobilized heavy metals was evaluated following the U.S. Environmental Protection Agency (EPA) Toxicity Characteristic Leaching Procedure (TCLP; EPA Method 1311). Based on preliminary evaluations (Section 7.1 of TCLP), extraction fluid no. 1 (Section 5.7.1 of TCLP) was used for the leaching test. Metal concentrations in the TCLP leachates of zeolite products were measured by ICP-MS, and the metal leaching efficiency was calculated by eq 6:
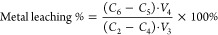
6where *C*_5_ and *C*_6_ (ppm) are metal concentrations in solution before and after the TCLP test, respectively, and *V*_3_ and *V*_4_ (L) are volumes of the oxalate filtrate that was used for zeolite synthesis and the solution that was used for TCLP test, respectively.

## 3 Results and Discussion

### 3.1 Characterizations of the MSWIA Sample

The concentrations of REE and non-REE of the studied MSWIA sample are shown in Table 1 and Table S1. Overall, this sample contains a total REE concentration of 391 ppm, with a notably high Nd content, likely due to lack of proper waste presorting processes in the U.S. and incorporation of electronic wastes into municipal solid wastes. The sample also contains high concentrations of non-REE such as Si, Ca, Al, Mg, Fe, and Na. SEM images show a highly heterogeneous morphology as expected, such as the presence of rod- and irregular-shaped particles and large clusters (Figure S1a,b). The particle size roughly ranged from 10 to 100 μm. XRD analysis reveals a 36.3 wt % of amorphous phase, and the major crystalline phases are calcium magnesium aluminum silicate (CaMgAl_2_SiO_7_), calcite (CaCO_3_), quartz (SiO_2_), and halite (NaCl) (Table 1, Figure S1c).

**Table 1:** Characteristics of the Raw MSWIA Sample^a^

**Major Element Composition by XRF (as Oxide wt %)**	
SiO_2_	38.2
CaO	22.6
Al_2_O_3_	13.5
MgO	5.3
Fe_2_O_3_	1.9
∑ Major oxides	81.5

**Phase Composition by XRD (wt %)**	
Amorphous	36.3
CaMgAl_2_SiO_7_	33.8
CaCO_3_ (Calcite)	20.6
SiO_2_ (Quartz)	5.8
NaCl (Halite)	3.5

**REE Concentration (ppm)**	
Sc	3.12 ± 0.25
Y	12.70 ± 0.73
La	24.58 ± 1.23
Ce	44.58 ± 3.48
Pr	65.59 ± 3.76
Nd	219.78 ± 14.53
Sm	2.08 ± 0.34
Eu	0.72 ± 0.13
Gd	2.26 ± 0.24
Tb	0.05 ± 0.01
Dy	14.01 ± 0.88
Ho	0.04 ± 0.01
Er	0.84 ± 0.14
Tm	BDL
Yb	0.69 ± 0.12
Lu	BDL
∑ REE	391.06 ± 22.57

a BDL: below detection limit.

### 3.2 Extraction of REE via Citrate Leaching

Based on the preliminary leaching experiments on MSWIA of three different particle sizes, extraction of REEs was remarkably more efficient as the particle size decreased (Figure S1d), which was expected because of the higher accessible surface area for reactions. Hence, MSWIA with a particle size below 106 μm was used in this study as a proof-of-concept.

In the control group (pH 4, no citrate), negligible leaching of both non-REE and REE was observed within 24 h (Table S2). In the presence of citrate, the kinetics results showed that REE leaching efficiency reached a steady state at ∼4 h (Figure S2). The solution pH increased slightly from 4.0 to 4.5. The leaching efficiency of individual REE varied from 20% to ∼100%. In addition to REE, citrate also concurrently extracted other metals such as Mg, Ca, Al, and Fe with similar kinetics (Figure S3). Thus, a reaction time of 4 h was used for all subsequent citrate extraction experiments. Since depletion of citrate could be a major cause for the plateaued leaching efficiency after 4 h, a cyclic experiment was conducted with 6 cycles and 50 mM of citrate at each replenishment. As shown in Figure S4, the total REE leaching efficiency was improved from 56.3% (1 cycle) to 80.7% (4 cycles), but additional cycles only showed negligible improvements (82.1% at 6 cycles).

**Figure 1:**
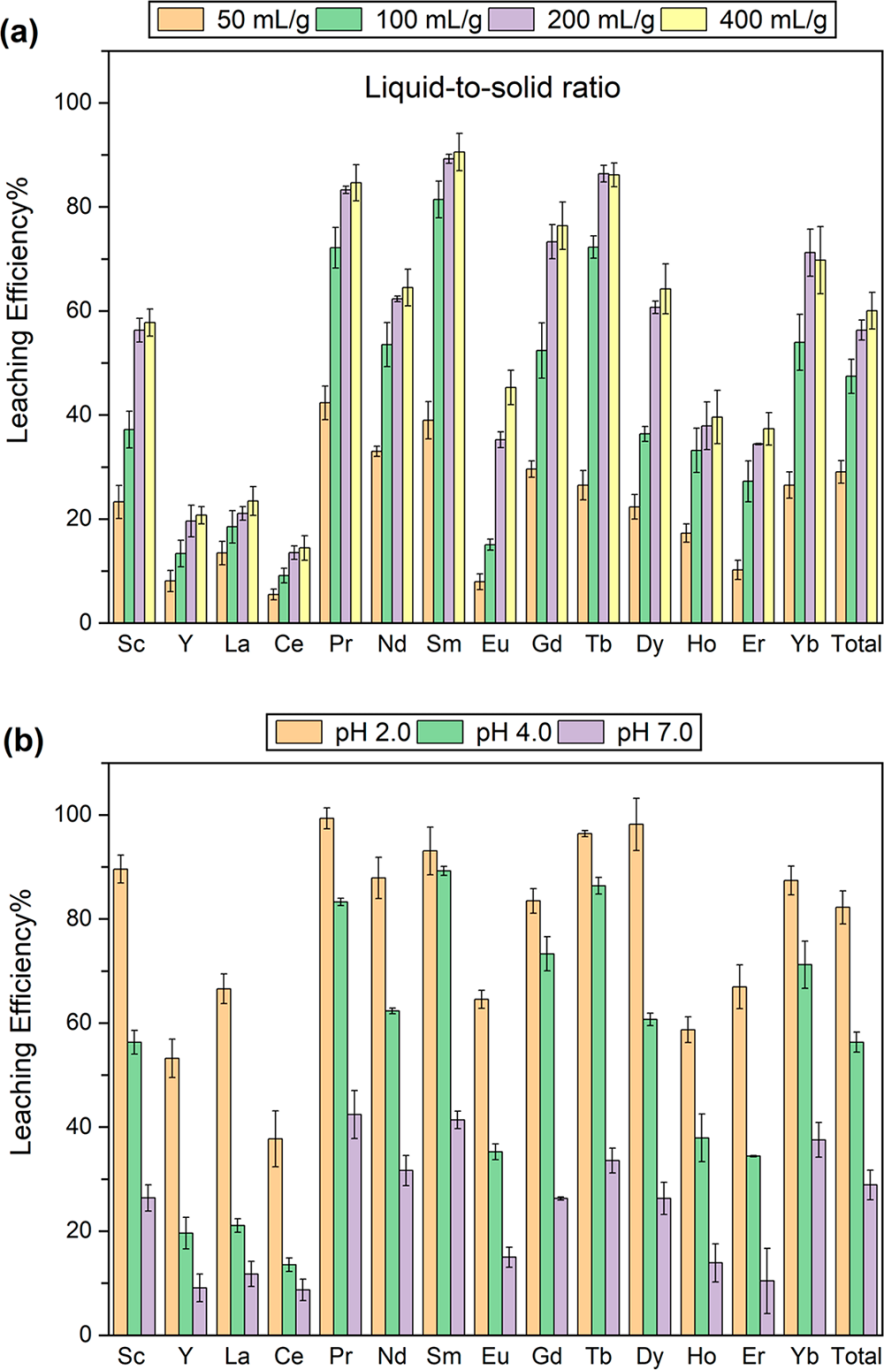
Efficiency of citrate-assisted REE leaching from MSWIA. (a) Effect of liquid-to-solid ratio (50–400 mg/L) (50 mM citrate, pH 4.0, 4 h, duplicate). (b) Effect of pH (2–7) (50 mM citrate, liquid-to-solid ratio 200 mL/g, 4 h, duplicate).

REE leaching efficiency at different liquid-to-solid ratios (50–400 mL/g) (Figure 1a) showed that an increase from 50 to 200 mL/g markedly enhanced REE leaching, likely due to more efficient metal–ligand complexation at higher citrate concentration. Further increasing the ratio from 200 to 400 mL/g did not result in significant improvements, possibly due to the saturation of surface complexation that was limited by effective surface contact between MSWIA particles and citrate molecules. We thus chose 200 mL/g as the optimal liquid-to-solid ratio in this system.

REE leaching efficiency was significantly enhanced by acidic pH over the studied pH range 2–7 (Figure 1b). The total REE leaching efficiency increased from 28.9% at pH 7 to 56.3% at pH 4 and 82.2% at pH 2. For Pr, Nd, Sm, and Dy, the leaching efficiency was above 90% at pH 2 but dropped to below 40% at pH 7. For most of the REE, over 60% was extracted at pH 4, a relatively mild condition. Interestingly, the leaching efficiencies of Y, La, and Ce were only around 20% at pH 4. According to Pearson’s hard and soft acids and bases (HSAB) theory, Y, La, and Ce are considered “softer” Lewis acids compared to other REE because they have larger ionic radii and lower charge density and are thus more polarizable. They tend to bind more strongly to soft Lewis bases (more polarizable donor molecules) than strong Lewis bases (e.g., citrate).^45^

The enhancive effect of low pH on ligand-promoted metal extraction can be explained by the proton-assisted mechanisms in three steps: (1) protons rapidly adsorb onto the mineral surface and protonate the metal, which polarizes and weakens the metal–oxygen bonds; (2) the metal–oxygen bonds cleave, and metal cations detach from the mineral (rate-limiting step); (3) the detached metal cations complex with ligands and enter the aqueous phase, leaving the initial metal site charge balanced.^46,47^ Conventional hydrometallurgy methods use concentrated inorganic acids to create an extremely acidic condition and often at an elevated temperature to facilitate step 1.^48,49^ It is noteworthy that the dissolution rate of minerals has a negative correlation with the concentration of free metal ions in solution.^50^ Since metals that are extracted by inorganic acids mostly exist as free cations in solution (e.g., M^3+^, M(OH)^2+^, and M(OH)_2_^+^), the build-up of metal ions as dissolution proceeds inhibits further dissolution.^50^ This limitation can be overcome by using organic acids, which promote the detachment of metal cations by forming soluble metal–ligand complexes (step 3) instead of free metal cations and thus do not impede step 2.

### 3.3 Selective Concentration of REE via Oxalate Coprecipitation

**Figure 2:**
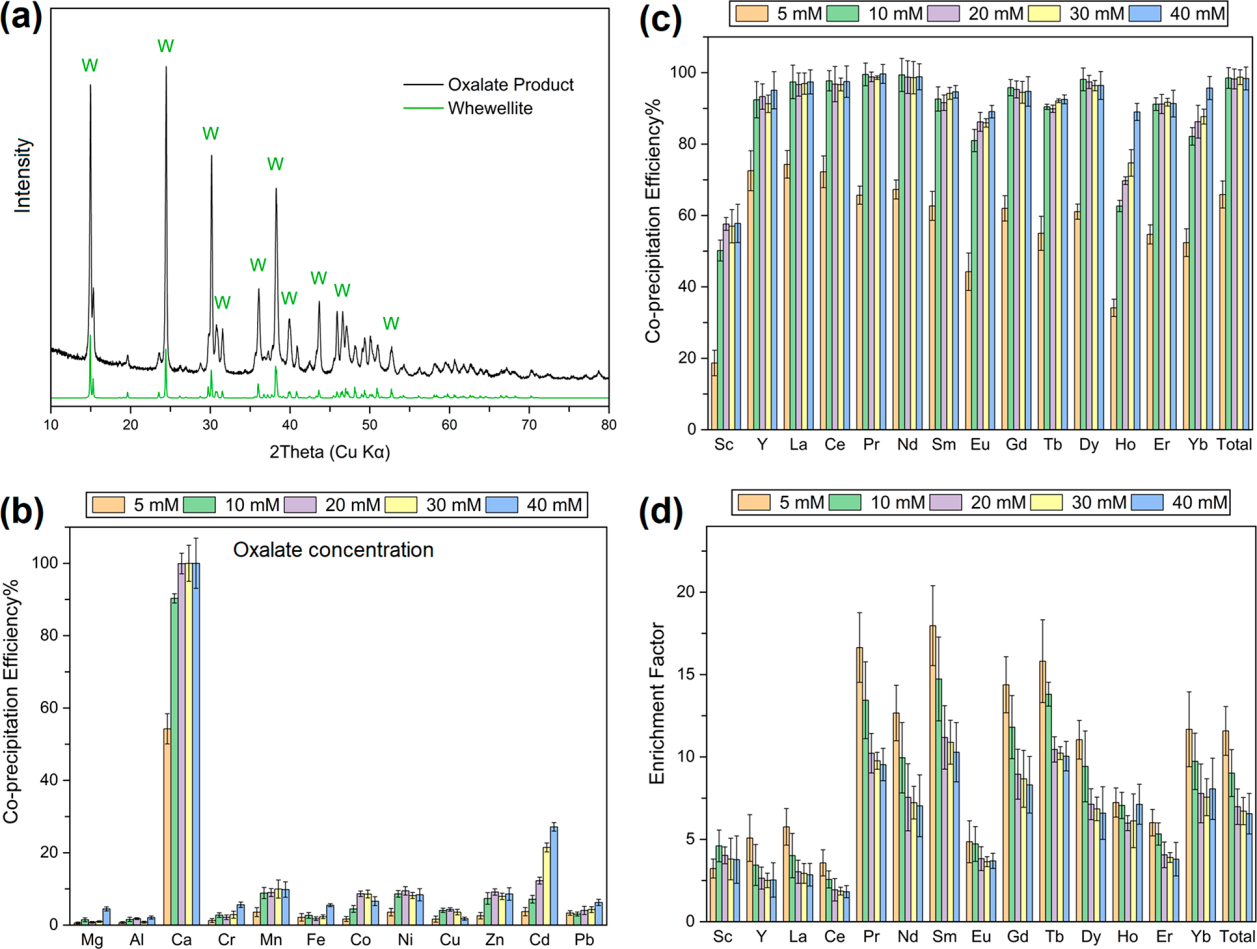
(a) XRD pattern (including the reference pattern of whewellite) of oxalate product. Coprecipitation efficiency of (b) non-REE and (c) REE at different oxalate concentrations (5–40 mM). (d) Corresponding enrichment factor of REE (30 min reaction time, using the citrate leachate from leaching experiments with 50 mM citrate, pH 4, liquid-to-solid ratio 200 mL/g, 4 h, duplicate).

The concentrations of non-REE and REE in citrate leachate are presented in Table S3. Besides REE, the citrate leachate also contained elevated levels of interfering metals such as Mg, Al, Fe, and Zn (Table S3). Fe and Al are of particular concern owing to the same oxidation state as REE and higher concentrations than other metals. In order to separate REE from non-REE, oxalate at various concentrations was added to the citrate leachate. Within a short reaction time of 30 min, we observed the formation of white precipitates. The solution slightly increased from 4.5 to 5.1. SEM images revealed the characteristic near-rhombic shape of the whewellite crystals (Figure S5a,b). Based on XRD analysis, the precipitate is predominantly the Ca-oxalate mineral whewellite (CaC_2_O_4_·2H_2_O) (Figure 2a). Some particles are still irregularly shaped with round edges, possibly due to incomplete nucleation and crystal growth within such a short reaction time. By comparing the concentrations of non-REE before and after oxalate precipitation, Ca^2+^ was preferentially precipitated with oxalate (Figure 2b), agreeing with previous thermodynamic modeling results that Ca-oxalate is the only oversaturated phase (saturation index >0) as the Ca^2+^ concentration is dramatically higher than REE and other non-REE, and its reaction with oxalate is thermodynamically favorable.^39^

Since the citrate leachate contained 11.7 ± 0.8 mM Ca^2+^, only around half of it precipitated with 5 mM oxalate, whereas over 90% of Ca^2+^ was precipitated in the presence of 10 mM oxalate (Figure 2b). Non-REE remained mostly dissolved in the leachate (<10% precipitation), likely because of their much smaller ionic radii (∼0.53–0.75 Å) compared to Ca^2+^ (1.12 Å) (Table S4).^51^ According to Goldschmidt’s rules, such a large size difference causes incompatibility and limits the substitution at Ca sites. In contrast, REE coprecipitation with Ca-oxalate was remarkably efficient. Increasing oxalate concentration from 5 to 10 mM improved the total coprecipitation% of REE from ∼65% to almost 100%, and then it reached a plateau as the oxalate concentration further increased (Figure 2c). Trivalent REE cations generally form an 8-fold coordination similar to that of the Ca site in whewellite, and their ionic radii (∼0.99–1.16 Å) are very similar to that of Ca^2+^. Due to these attributes, REE substitution in Ca-oxalate is highly favored with an equilibrium constant (*K*_eq_) typically greater than 10^5^.^52^ As an exception, the apparently lower coprecipitation % of Sc is likely due to its smaller ionic radius (0.87 Å) compared to that of other REE and Ca^2+^.

The enrichment of REE during the oxalate precipitation step (i.e., enrichment factor) was positively related to the coprecipitation efficiency of REE but inversely related to the total mass of the solid precipitates. Using 5 mM oxalate achieved the highest enrichment factor (Figure 2d). Even though the enrichment factor in the presence of 10 mM oxalate was slightly lower than that with 5 mM oxalate, the ∼35% increase in the total REE coprecipitation % was significant. The enrichment factor reached a plateau from 20 to 40 mM oxalate, because the mass of Ca-oxalate and REE coprecipitation efficiency both remained unchanged. The total enrichment factor of REE with 10 mM oxalate reached 9.07, which is significantly higher than other enrichment methods such as magnetic separation (1.01–1.16) and density separation (1.18–3.58).^53−55^ Furthermore, the total REE concentration in the oxalate product reached >3500 ppm (Table S5). The oxalate product can be used as a concentrated feedstock for the downstream production of REE-oxide via calcination or REE-concentrate via acid digestion. With easy operation and a short reaction time, oxalate coprecipitation holds appealing advantages in selective purification and concentration of REE. Note that since the log β of the REE-oxalate complex (∼5.8–7.1) is generally lower than that of the REE-citrate complex (∼6.7–11.8),^56^ precipitation of REE in the citrate leachate relies on the presence of Ca-oxalate. Thus, this system should be effective for other Ca-rich feedstocks as well, including natural REE minerals, though additional input of Ca may be needed for feedstocks with a very low Ca content (e.g., electronic waste).

### 3.4 Waste Upcycling and Heavy Metal Immobilization via Zeolite Synthesis

The citrate extraction step produces a solid waste byproduct (citrate residue) with abundant Si and Al, and the oxalate coprecipitation step produces a liquid wastewater (oxalate filtrate) containing elevated levels of non-REE heavy metals (Table S7). To minimize secondary pollution, zeolite synthesis was conducted to upcycle these waste byproducts. Previous studies have shown that higher temperature and alkaline conditions are favorable for zeolite nucleation and crystal growth.^57^ Here, we applied a low-temperature hydrothermal method to reduce the energy and chemical input. The reaction temperature was 150 °C, and the initial NaOH concentration was varied at 5.0 M (initial pH = 13.9 ± 0.1) and 0.5 M (initial pH = 12.4 ± 0.1) (hereafter denoted as zeolite-A and zeolite-B, respectively).

**Figure 3:**
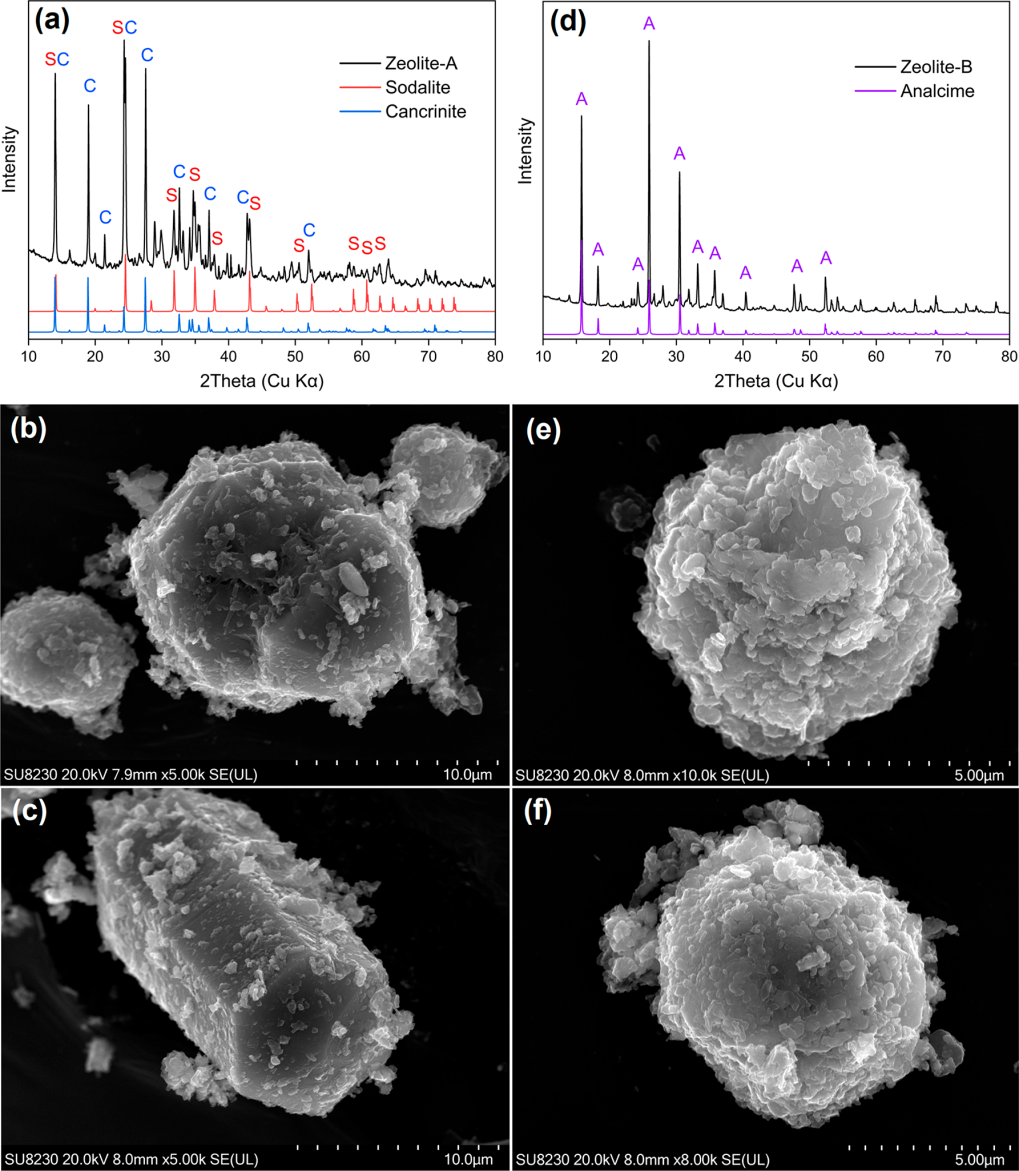
XRD patterns (including reference patterns of identified phases: S, sodalite; C, cancrinite; A, analcime) and SEM images of zeolites synthesized in the presence of (a–c) 5 M NaOH (Zeolite-A) and (d–f) 0.5 M NaOH (Zeolite-B). Citrate residue conditions: 50 mM citrate, liquid-to-solid ratio 200 mL/g, pH 4.0, 4 h. Oxalate filtrate conditions: 10 mM oxalate, 30 min. Zeolite synthesis conditions: liquid-to-solid ratio 50 mL/g, 150 °C, 12 h.

XRD analysis confirmed the successful synthesis of zeolite group minerals, with the specific phases varying with alkaline dosage and initial pH (Figure 3a,d, Table S6). The crystalline products in zeolite-A consisted of 51.1% sodalite and 48.9% cancrinite, whereas zeolite-B contained almost completely analcime. Such a phase difference was observed under SEM. For zeolite-A, most particles have an average size of ∼5–10 μm and relatively clear edges that resemble a near-hexagonal shape (Figure 3b). Notably, we also observed some well-crystallized hexagonal prismatic crystals with lengths of more than 10 μm (Figure 3c). The zeolite-B phases, on the other hand, contain mostly clusters of crystal agglomerates with an average size of ∼5 μm and do not form any clear shapes (Figure 3e,f). More SEM images of the zeolite products are included in Figure S6. Elemental mappings and point spectra of selected zeolite particles confirmed the dominant presence of Si, Al, O, and Na (Figures S7 and S8), agreeing well with the XRD results (Table S6). These results suggest that pH indeed plays an important role in the zeolite phase formation, degree of crystallinity, and crystal morphology. A higher pH favors a better nucleation and crystal growth. Since the zeolite filtrate was still highly alkaline (pH >11) and contained abundant Si and Al, reusing it for multiple cycles of zeolite synthesis is possible.

**Figure 4:**
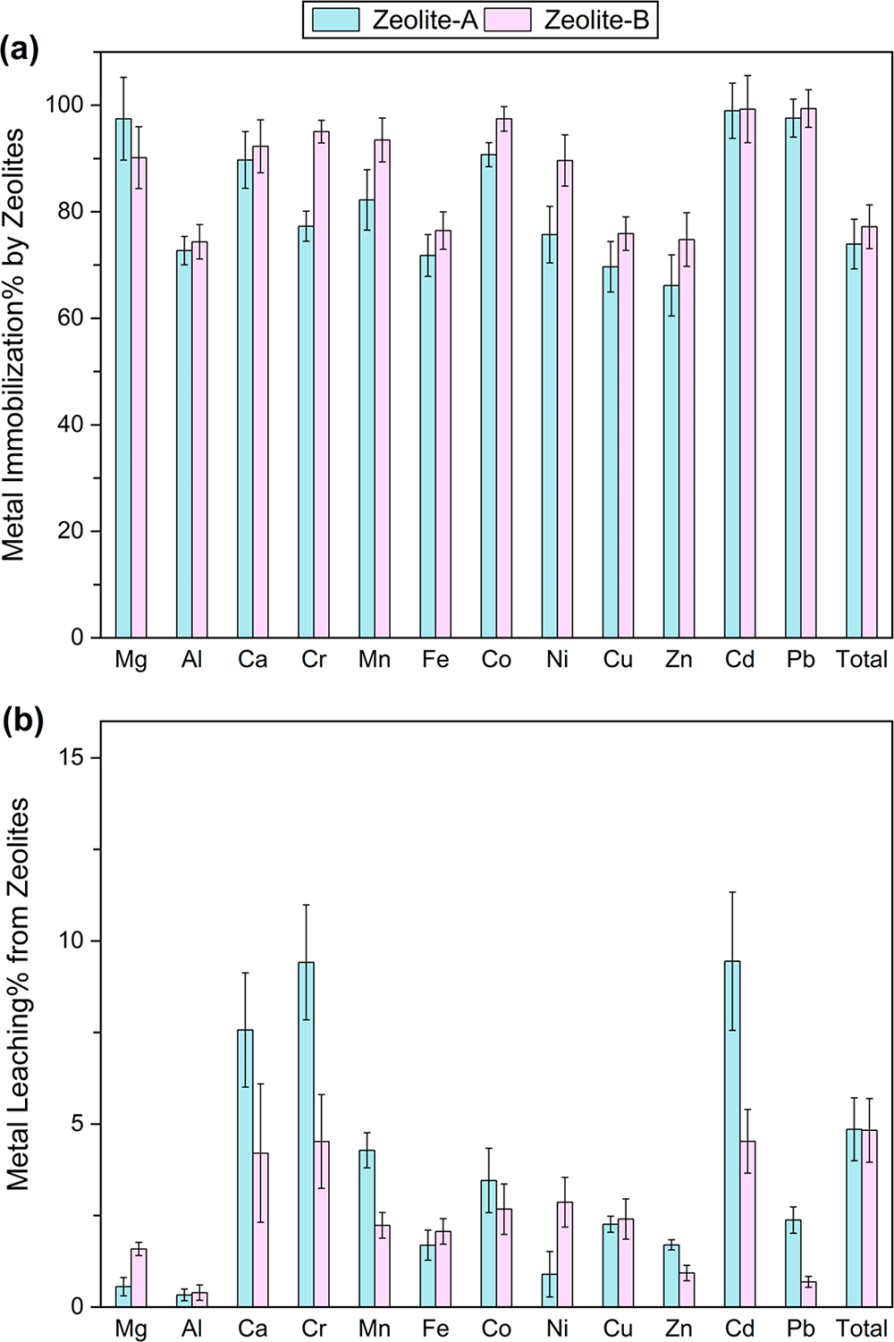
(a) Immobilization of non-REE metals in the synthesized zeolite products. (b) Percentage of non-REE metals leached from the zeolite products during TCLP test. All experiments in duplicate.

A main benefit of zeolite synthesis is the removal of toxic heavy metals from wastewater. We compared the concentrations of non-REE in the aqueous phase before and after zeolite synthesis (eq 5). The overall metal immobilization efficiency by zeolite-A and zeolite-B was 77.2% and 73.9%, respectively (Figure 4a). More than 80% of toxic heavy metals (e.g., Cr, Co, Ni, Cd, and Pb) were removed by zeolites, with a total of <50 ppb remaining in the wastewater (Table S7). While zeolites also immobilized ∼70% of Mg, Al, Fe, Cu, and Zn, the remaining portions of these marketable metals (∼2–14 ppm) might still be worthy of recovery (e.g., solvent extraction, ion exchange, electrodeposition). We also conducted the EPA TCLP to evaluate the potential leaching of these immobilized heavy metals from zeolite, and the results demonstrated that the zeolite products only released minor amount of trace metals (<5% total) over 18 h (Figure 4b). Compared to raw MSWIA, the heavy metal concentrations in the TCLP leachates of zeolite products were drastically lower and almost met the EPA drinking water standards without any further treatment (Table S7). Negligible difference in metal leaching was observed for zeolite-A and -B, and thus, reducing the alkaline input did not significantly compromise the overall performance of zeolite products. The effective removal and stabilization of heavy metals by zeolite can be attributed to its stable framework, which can accommodate the incorporation or adsorption of various metal cations without destroying the crystal lattice.^58^ Another benefit of zeolite synthesis is waste volume reduction. From the initial MSWIA input to the final zeolite product, the overall solid volume reduction of this treatment system was 77.3% (eq 4), which is more desirable from the perspective of waste transportation and storage. The synthesized zeolites can be considered as a more stable and less hazardous option for waste storage and management compared to raw MSWIA.

### 3.5 System Sustainability

To better evaluate the sustainability of this treatment system based on the principles of green chemistry and engineering, important metrics are compared with existing literature on REE recovery from wastes (Table 2).^19,59−62^ A warmer highlight color indicates harsher reaction conditions, heavier environmental footprints, and lower system performance. We note that there are also many other promising technologies for REE recovery such as membrane-based processes.^63^ However, this section mainly focuses on comparison with previous hydrometallurgical methods because of their similar metrics. In addition, due to the lack of previous studies on REE recovery from MSWIA, the metrics in this study were compared to previous studies on CFA. Compared to previously reported technologies, our system features mild chemical and energy consumption, a low environmental impact, and a high overall system performance (extraction and separation).

**Table 2:**
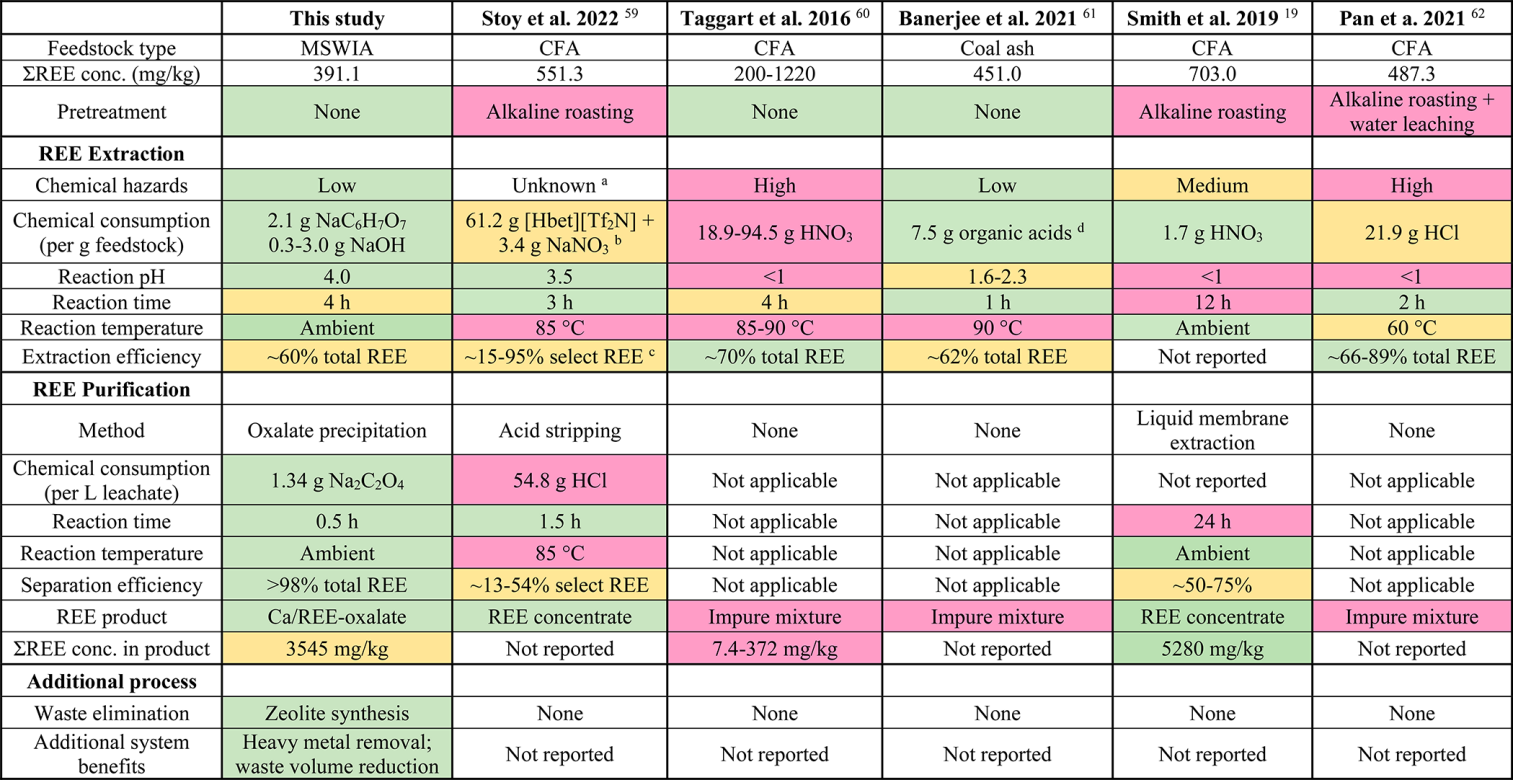
Comparison on the System Sustainability Metrics of This Study and Previous Works on REE Recovery

a The toxicity and environmental impact of betainium bis(trifluoromethylsulfonyl)imide ([Hbet][Tf_2_N]) have not been investigated.

b Calculated based on 1.531 g/cm^3^ density of [Hbet][Tf_2_N] as reported by Nockemann et al.^100^

c Select REE includes Sc, Y, La, Ce, Nd, Sm, Eu, Dy, and Yb.

d Organic acids tested include tartaric acid, malonic acid, lactic acid, citric acid, and succinic acid.

Pretreatment processes in many studies are designed to break down durable aluminosilicate matrixes (e.g., amorphous glass phase) and release encapsulated REE phases.^17,39^ These processes involve the addition of strong alkaline agents and heating at elevated temperatures (up to 450–800 °C),^19,62^ which often account for a substantial portion of the total operational cost. It is important to note that the efficient REE extraction in our study without any pretreatments might have benefited from the relatively low Si content in MSWIA (Table 1 and Table S1) compared to CFA (especially Class F).^64^ Given the high variability of MSWIA composition, comprehensive investigations are needed to further assess the applicability of this system on different MSWIA, such as those from different incineration facilities and sources, burning conditions, separation steps, etc. While conventional hydrometallurgy processes require the use of concentrated strong inorganic acids to improve the overall REE extraction efficiency,^11,12^ our results demonstrated a comparable performance by dilute citrate under a mildly acidic condition. For example, Taggart et al. extracted ∼70% of total REE from CFA in 4 h using 15 M HNO_3_ at around 90 °C.^60^ We demonstrated that 50 mM of citrate extracted ∼60% of total REE in 4 h at pH 4 without any thermal input. The use of non-toxic and biodegradable citrate over mineral acids also favors secondary pollution control. In terms of cost, citrate can be readily produced via microbial fermentation of *Aspergillus* or *Candida* at an industrial scale with a reasonable price.^65^

For REE purification, Stoy et al. transfers ∼13–54% of extracted REE from ionic liquid to the aqueous phase using HCl as a stripping agent at 85 °C.^59^ Smith et al. selectively separates ∼50–75% REE from interfering metals and obtained a total REE concentration of 5280 mg/kg in the product via liquid membrane extraction, but a long reaction time is required (24 h).^19^ In this study, the use of oxalate coprecipitation is proven efficient (>98% REE precipitation) and fast (30 min). Although the oxalate product contains a high Ca content, trivalent REE can be easily separated from Ca using commercially available technologies, including ion exchange and electrodialysis. Notably, we emphasize that control of waste byproducts is vital in the perspective of system sustainability, yet very few studies have attempted to address that. Zeolite synthesis in this study offers the dual benefits of waste stream decontamination and waste volume reduction, which allows a less demanding post-treatment and safer storage of waste residue. The reaction temperature of the zeolite synthesis is also relatively mild and can be self-sustained in situ by the thermal energy released from waste incineration. In addition, since the zeolite filtrate is still basic (pH >11) and contains abundant Si and Al, wastewater reuse over multiple cycles of zeolite synthesis is possible, which requires future studies to verify.

## 4 Conclusions

In this study, we applied a three-step modular treatment system to recover REE from MSWIA and minimize secondary wastes. Citrate as a non-hazardous organic ligand at a dilute concentration extracted 82.2% of total REE at pH 2.0 and 56.3% at pH 4.0. Addition of oxalate achieved a near-complete (98.5%) coprecipitation of REE with an enrichment factor of 9.1 under optimal conditions, while non-REE mostly remained in the aqueous phase. The total REE concentration in the purified solid product reached 3545 ppm. To eliminate the generated waste byproducts, citrate residue and oxalate filtrate were upcycled to synthesize zeolite via a low-temperature hydrothermal method. The zeolite products have a ∼80% reduced volume compared to raw MSWIA, which is particularly favorable for waste transportation and storage. During zeolite synthesis, ∼75% of heavy metals in the wastewater were removed and stabilized by the zeolite products with <5% leaching during TCLP. A lower NaOH dosage led to a decreased crystallinity and crystal size of zeolite but did not significantly compromise the heavy metal immobilization efficiency. The waste upcycling process substantially reduced the need for wastewater post-treatment, and the zeolites could be stored as a less hazardous and more cost-effective alternative to MSWIA. Overall, this study demonstrated an easy-to-operate technology for secondary REE production and concurrent waste management. The modular design of this system could allow a high flexibility for modification and could accommodate different needs, which is ideal for large-scale applications. On the other hand, this treatment system currently has a few limitations that require future investigations, such as strategies to reduce chemical costs (e.g., reducing oxalate dosage or recycling oxalate) and simultaneous recovery of other valuable metals (e.g., Mg, Al, Fe, Cu, Zn) from the oxalate filtrate, as well as techno-economic analysis, system upscaling, and evaluation of other feedstocks.

## Supporting Information Available

Experimental details about chemicals, MSWIA samples, XRF spectrometry, ICP-MS, SEM-EDX, and XRD; tables of compositions of non-REE elements in MSWIA, leaching efficiencies, concentrations of non-REE and REE in citrate leachate and of REE in raw MSWIA, ionic radii, mineralogical characteristics of synthesized zeolites, concentrations of trace metals in the TCLP leachates; figures of SEM images, XRD patterns, leaching efficiencies, elemental maps and point spectra (PDF)
