# Supplementary material for: Rare Earth Elements Recovery and Waste Management of Municipal Solid Waste Incineration Ash
Source: ACS Sustain Resour Manag. 2023 Nov 29;1(1):17–27. doi: 10.1021/acssusresmgt.3c00026 (PMC10840445; doi:10.1021/acssusresmgt.3c00026)
Supplement: Supplementary file 1 — rm3c00026_si_001.pdf [file rm3c00026_si_001.pdf]

Supporting Information

Rare Earth Elements Recovery and Waste Management of Municipal Solid Waste Incineration Ash

Yinghao Wen[1], Lei Hu[1], Anthony Boxleiter[2], Dien Li[3], Yuanzhi Tang[1*]

[1] School of Earth and Atmospheric Sciences, Georgia Institute of Technology, 311 Ferst Dr., Atlanta, GA 30332, USA

[2] Department of Geosciences, Georgia State University, 38 Peachtree Center Ave., Atlanta, GA 30303, USA

[3] Savannah River National Laboratory, Aiken, SC, 29808, USA

*Corresponding author:
Yuanzhi Tang
Email: yuanzhi.tang@eas.gatech.edu
Phone: 404-894-3814

Total 19 pages
6 texts
8 figures
7 tables

Text S1. Chemicals

Sodium dihydrogen citrate (>99%) was purchased from Alfa Aesar (Haverhill, USA). Sodium oxalate (>99%), hydrochloric acid (ACS grade, 36.5-38%), nitric acid (ACS grade, 68-70%), and sodium hydroxide pellets (>97%) were purchased from VWR (Radnor, USA). Glacial acetic acid (>99.7%) was purchased from Fisher Scientific (Hampton, USA). Indium internal standard (TraceCERT) and REE mix standard (TraceCERT) were purchased from Sigma-Aldrich (St. Louis, USA). Multi-element standard (Solution 2A) was purchased from SPEX CertiPrep (Metuchen, USA). Lithium tetraborate/metaborate/iodide flux blend "GF GF-65-5I" was purchased from Premier Lab Supply (Port St. Lucie, USA). Ultrapure deionized water (18.2 MΩ·cm) produced by a Barnstead Nanopure system (Thermo Fisher Scientific, Waltham, USA) was used in all solutions.

Text S2. Municipal solid waste incineration ash (MSWIA) samples

Municipal solid waste incineration bottom ash (MSWIA) samples were obtained from a Waste-to-Energy facility located in Northwest USA and size fractionated using standard testing sieves with size openings of 2 mm, 600 μm, and 106 μm. Prior to characterizations, MSWIA sample was grinded to fine powders using a pestle and mortar. The morphology of size-fractionated ash sample was examined using scanning electron microscopy (SEM). The mineral composition of the ash sample was examined using X-ray diffraction (XRD). The concentrations of trace metals in the ash samples were measured using X-ray fluorescence (XRF). A portion of the MSWIA sample was also digested using a Milestone Ultrawave Single Reaction Chamber microwave digestion system, and the digestate was analyzed for REE concentration using inductively coupled plasma mass spectrometry (ICP-MS).

Text S3. X-ray fluorescence spectrometry (XRF)

The elemental composition of major elements in the raw MSWIA sample were determined using a Rigaku Primus IV*i* wavelength-dispersive sequential X-ray fluorescence spectrometer (Tokyo, Japan). Dried MSWIA sample was fused as lithium borate glass discs. Lithium borate fusion was prepared with a dilution of 1:20 (sample:flux) using flux type GF-65-5I (65:35 blend of lithium tetraborate to lithium metaborate, 0.5 wt.% lithium iodide non-wetting agent purchased from Premier Lab Supply; Port St. Lucie, USA). Fusion was performed in Pt-Au crucibles using a two-position XRFuse2 fusion oven (Premier Lab Supply). The fused sample was cooled to glass discs in Pt-molds for XRF analysis. The operation voltage and current for XRF were 50 kV and 50 mA, respectively. Calibrations were monitored by drift corrections, standards check, and measurement of certified reference materials. Results were normalized to loss of ignition (LOI) that was measured by thermogravimetric analysis (TGA).

Text S4. Inductively coupled plasma mass spectrometry (ICP-MS)

The elemental concentrations in the raw ash sample digestate, citrate leachate, oxalate filtrate, and zeolite filtrate were measured using an Agilent 7500a ICP-MS (Santa Clara, USA). All calibration standards (0–400 ppb) and samples were prepared in 2% HNO_3 and spiked with 20 ppb of indium (In) as the internal standard. The instrument was tuned for high sensitivity, low isobaric interference ($MO^+/M^+ < 1\%$), and low doubly charged ions ($M^{++} < 2\%$). The elemental

isotopes that were measured include: ^{24}Mg, ^{27}Al, ^{40}Ca, ^{45}Sc, ^{53}Cr, ^{55}Mn, ^{56}Fe, ^{59}Co, ^{60}Ni, ^{63}Cu, ^{66}Zn, ^{89}Y, ^{111}Cd, ^{115}In, ^{139}La, ^{140}Ce, ^{141}Pr, ^{146}Nd, ^{147}Sm, ^{153}Eu, ^{157}Gd, ^{159}Tb, ^{163}Dy, ^{165}Ho, ^{166}Er, ^{169}Tm, ^{172}Yb, ^{175}Lu, and ^{208}Pb. Calibration standards were measured after every 30 samples to ensure accuracy.

Text S5. Scanning electron microscopy coupled with energy dispersive X-ray spectroscopy (SEM-EDX)

The morphology of raw MSWIA sample, oxalate precipitates, and zeolite products was examined using a Hitachi SU8320 SEM (Hitachi, Japan) coupled with Oxford X-Max^{N} EDX (Oxford Instruments, U.K.). Samples were mounted on a carbon tape. One layer of carbon coating (34 nm thickness) was applied to all samples using a Quorum Q150V Plus coater (Quorum Technologies, U.K.). SEM images were taken at 20 kV, 10 µA, and a working distance of 8.0 mm. Elemental maps and point spectra were taken at 20 kV, 10 µA, and a dwell time of 200 ms.

Text S6. X-ray diffraction (XRD)

The mineralogy and phase identification of the raw ash sample, oxalate precipitates, and zeolite products were analyzed by XRD using a Panalytical Empyrean multipurpose diffractometer equipped with a PIXcel 3D-Medipi detector (Malvern, UK). Data were collected from 10 to 80 ˚2θ with a step size of 0.03 ˚2θ. Cu Kα (1.5406Å) was used as the radiation source. The operation voltage and current were 45 kV and 40 mA, respectively. The collected XRD patterns were processed using HighScore and refined using Rietveld methods with phase ID references patterns sourced from the International Centre for Diffraction Data (ICDD) database.

Table S1. Compositions of non-REE elements in raw MSWIA sample measured by XRF.

Element	Elemental Concentration (ppm)
Na	32983.3
Mg	11741.2
Al	71466.1
Si	178440.6
P	8794.0
S	7088.1
Cl	4446.0
K	11165.5
Ca	161850.0
Ti	5987.5
Cr	294.2
Mn	1127.5
Fe	36916.0
Co	84.5
Ni	157.2
Cu	5648.0
Zn	11360.1
Sr	778.0
Cd	32.5
Ba	2095.8
Pb	2525.0
Σ non-REE elements	555066.4

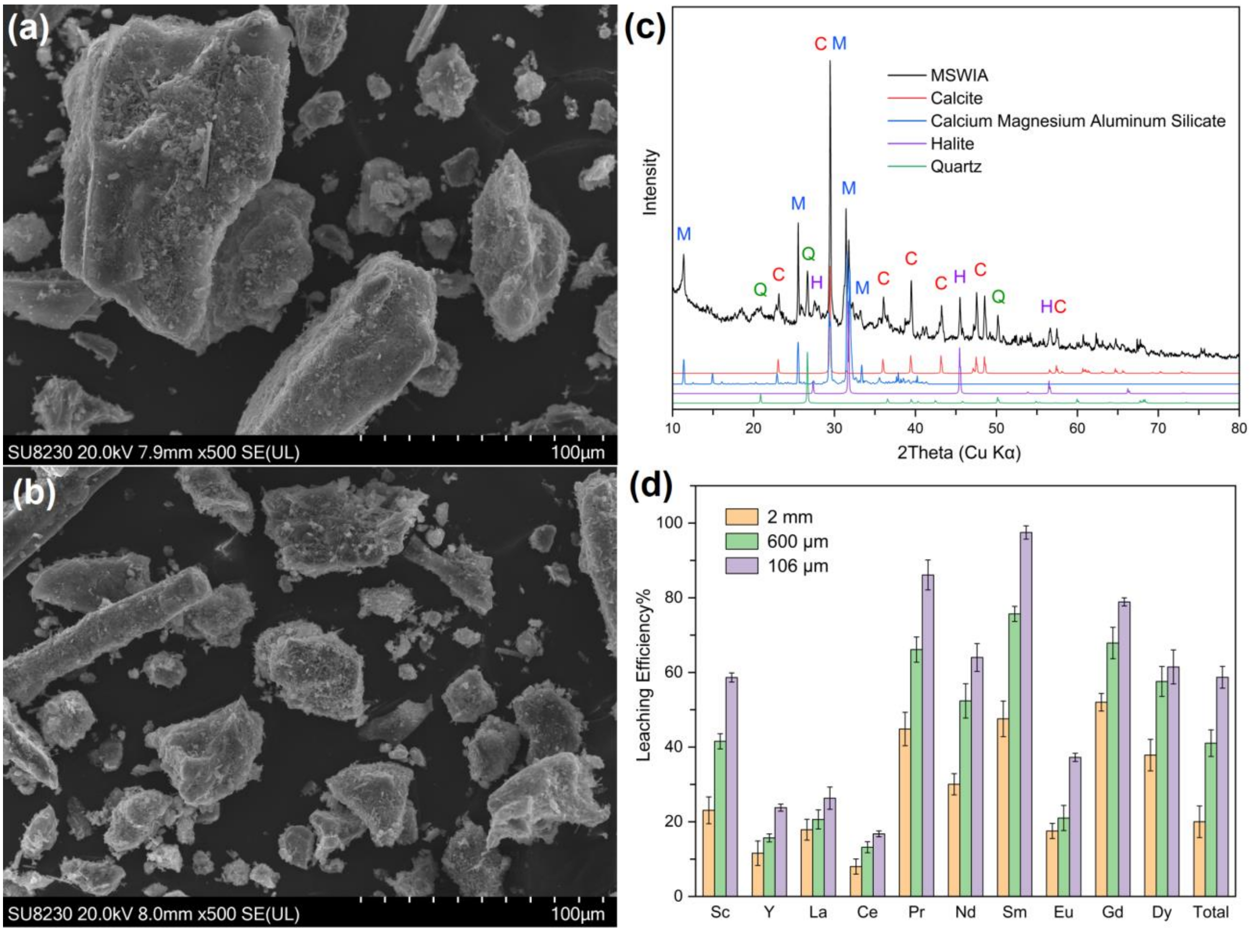

Figure S1. (a-b) SEM images and **(c)** XRD patterns (including reference patterns of identified mineral phases; C: calcite; M: calcium magnesium aluminum silicate; H: halite; Q: quartz) of 106 µm MSWIA; **(d)** leaching efficiency of REEs from MSWIA of different particle sizes (50 mM citrate, liquid-to-solid ratio 200 mL/g, pH 4.0, 24 h, duplicate.).

Table S2. Leaching efficiencies of non-REE and REE without citrate at pH 4 within 24 h.

Element	Leaching efficiency%
Mg	1.11
Al	0.43
Ca	0.68
Cr	0.13
Mn	0.08
Fe	0.77
Co	1.35
Ni	1.76
Cu	0.69
Zn	1.16
Cd	1.62
Pb	0.35
Sc	0.44
Y	0.67
La	0.95
Ce	0.24
Pr	0.37
Nd	0.19
Sm	0.66
Eu	1.24
Gd	0.88
Tb	0.18
Dy	0.43
Ho	1.48
Er	0.82
Yb	1.55

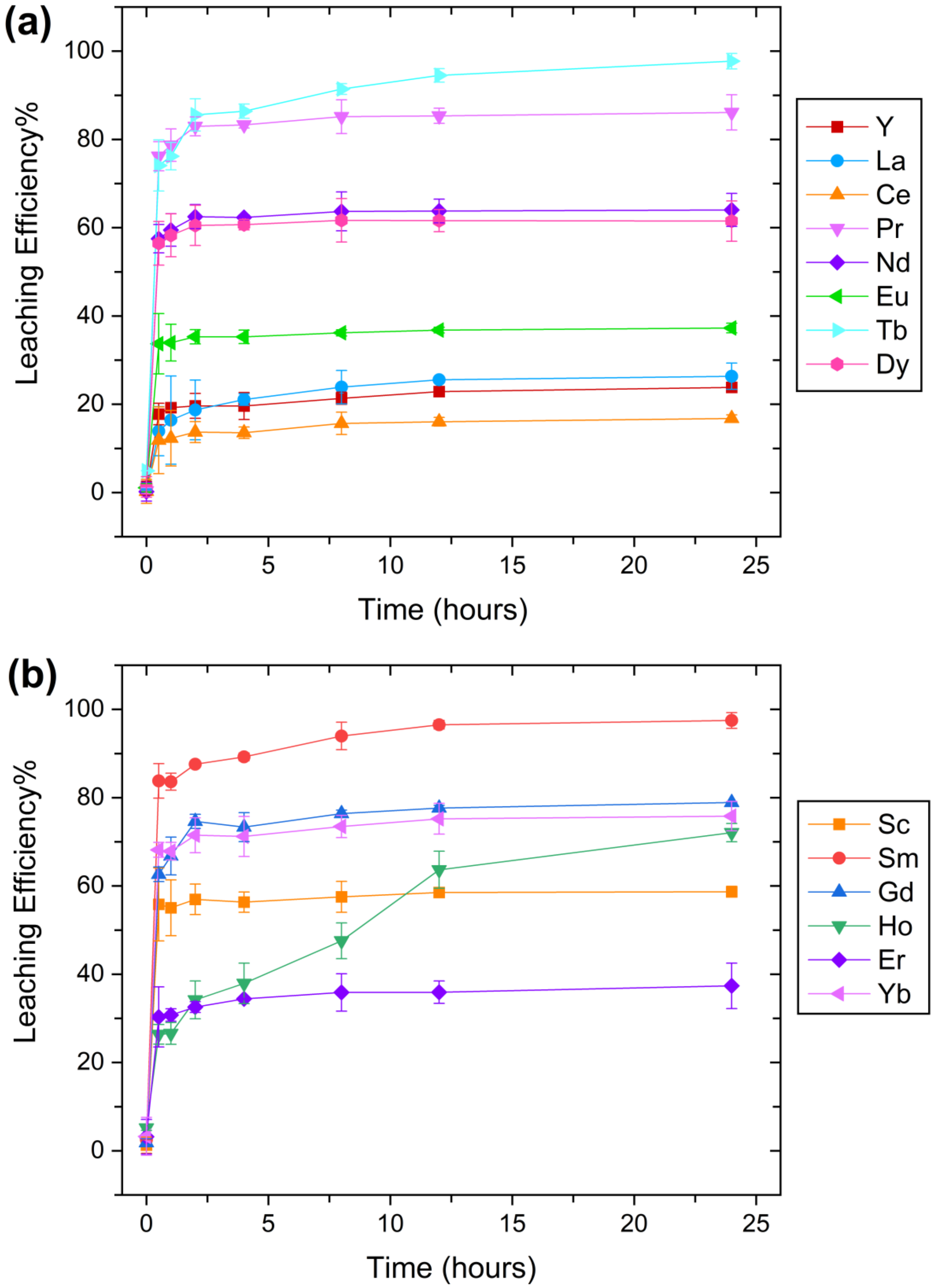

Figure S2. (a-b) The leaching efficiency of REE from MSWIA by citrate over time (50 mM citrate, liquid-to-solid ratio 200 mL/g, pH 4.0, 24 h, duplicate).

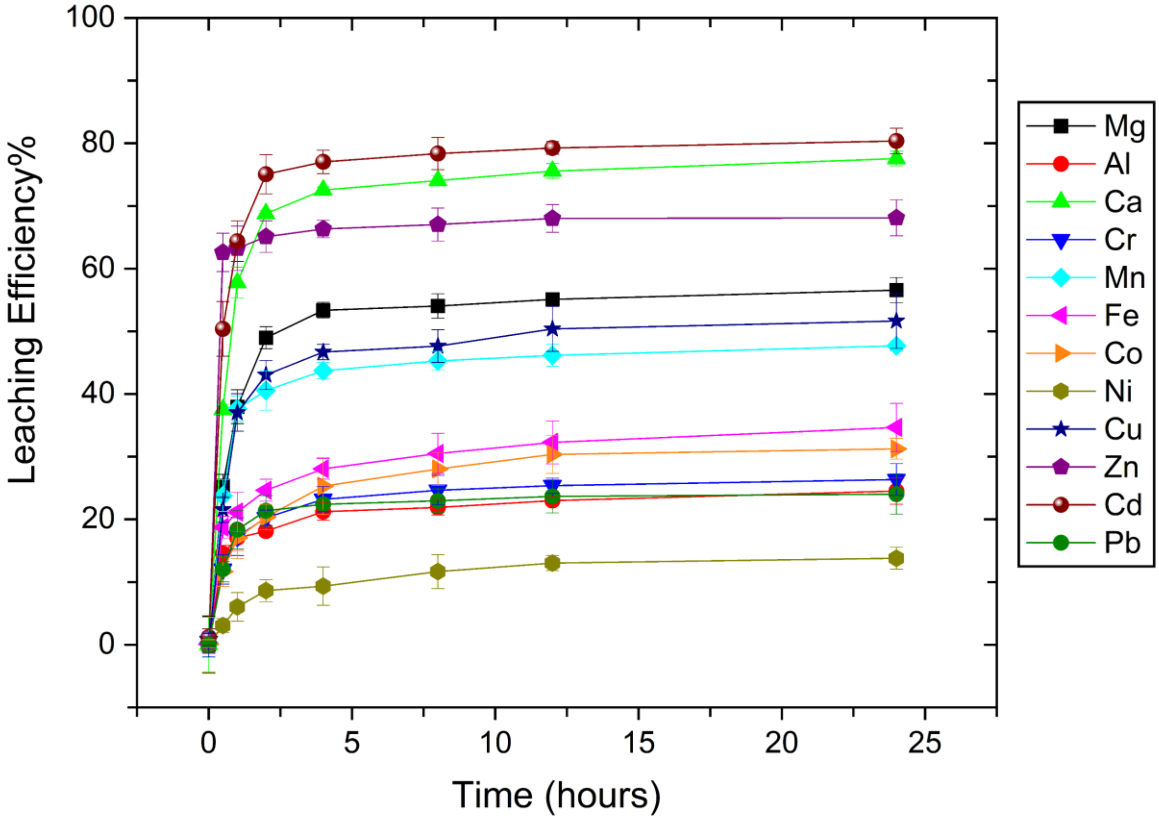

Figure S3. The leaching efficiency of non-REE metals from MSWIA sample by citrate over time (50 mM citrate, liquid-to-solid ratio 200 mL/g, pH = 4.0, 24 h, duplicate).

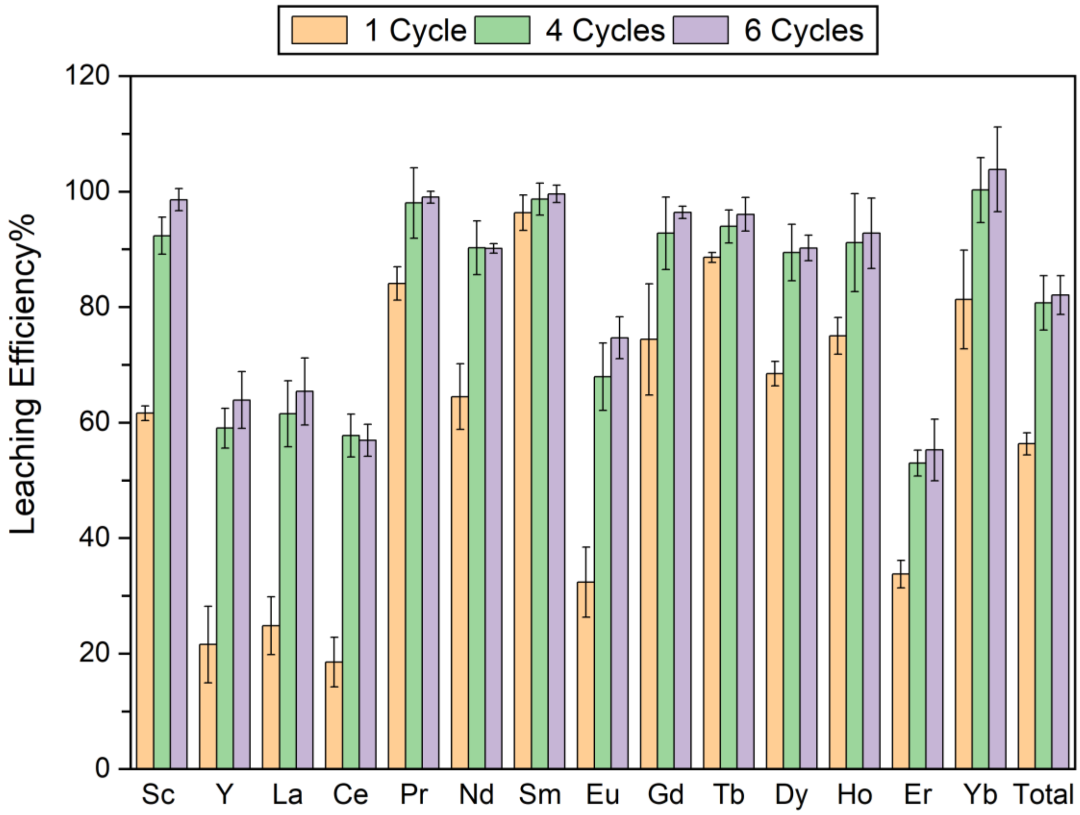

Figure S4. Leaching efficiency of REE from MSWIA sample over multiple cycles (50 mM citrate in each cycle, pH 4.0, liquid-to-solid ratio 200 mL/g, 4 h per cycle, duplicate).

Table S3. Concentrations of non-REE and REE in citrate leachate (50 mM citrate, pH 4.0, liquid-to-solid ratio 200 mL/g, 4 h).

**Non-REE**	**Concentration (ppm)**
Mg	35.12 ± 2.23
Al	84.05 ± 5.78
Ca	737.66 ± 31.52
Cr	0.18 ± 0.03
Mn	5.09 ± 0.55
Fe	45.96 ± 3.77
Co	0.11 ± 0.02
Ni	0.12 ± 0.02
Cu	12.15 ± 1.04
Zn	35.56 ± 2.66
Cd	0.05 ± 0.01
Pb	3.28 ± 0.42
Σ non-REE	978.04 ± 51.21
**REE**	**Concentration (ppb)**
Sc	9.14 ± 1.05
Y	15.11 ± 1.42
La	32.37 ± 1.87
Ce	37.42 ± 2.12
Pr	282.44 ± 7.51
Nd	703.53 ± 12.63
Sm	10.16 ± 0.99
Eu	1.34 ± 0.21
Gd	8.93 ± 0.91
Tb	0.26 ± 0.04
Dy	43.08 ± 2.68
Ho	0.14 ± 0.02
Er	1.57 ± 0.23
Yb	2.62 ± 0.35
Σ REE	1148.21 ± 28.64

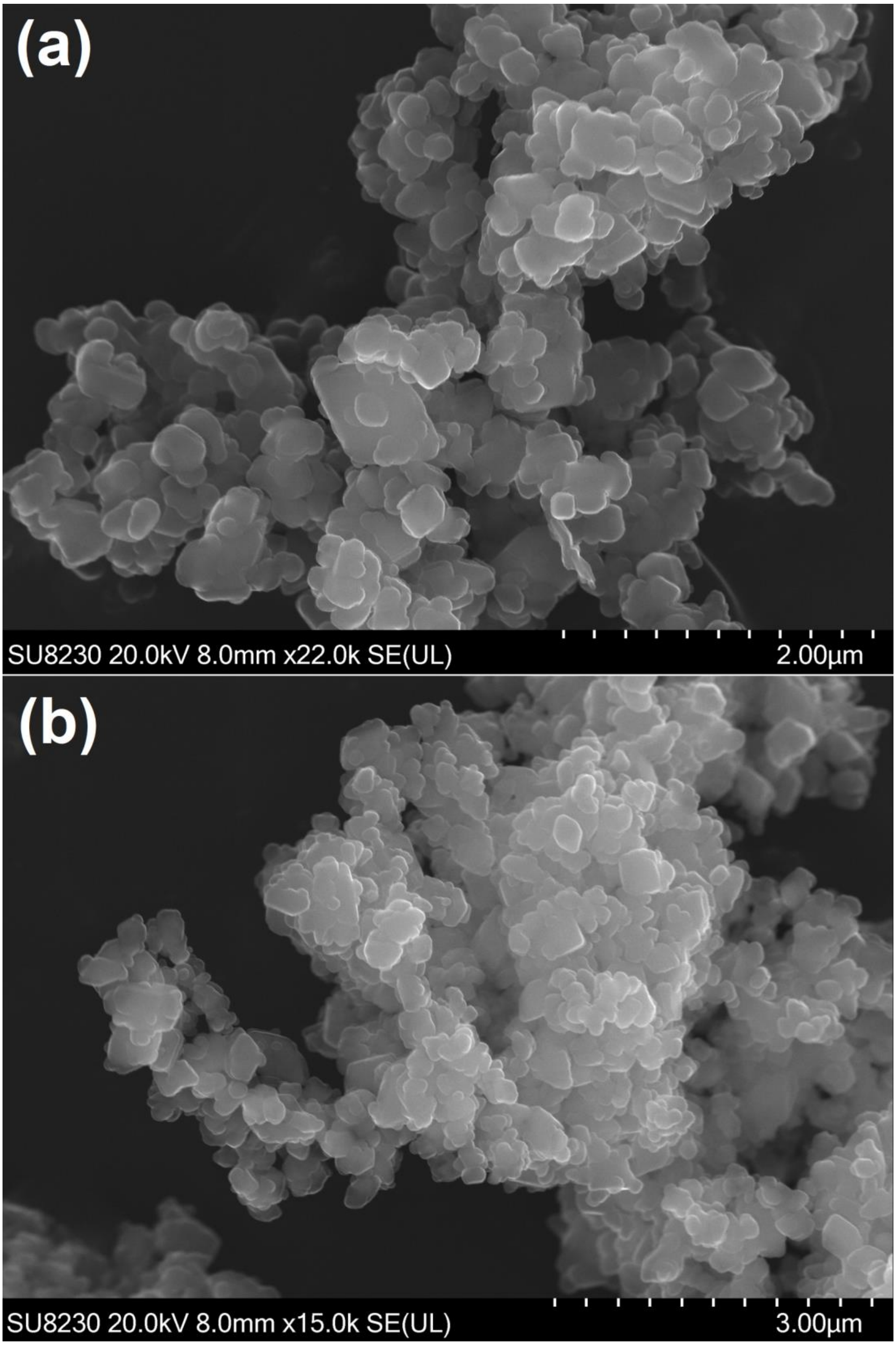

Figure S5 a-b. SEM images of oxalate product

Table S4. Ionic radii of non-REE elements and REE in their most common oxidation states and coordination numbers (retrieved from Shannon 1976).[1]

Element	Oxidation State	Coordination Number	Ionic Radius (Å)
**Non-REE**			
Mg	+2	VI	0.72
Al	+3	VI	0.54
Ca	+2	VIII	1.12
Cr	+3	VI	0.62
Mn	+4	VI	0.53
Fe	+3	VI	0.55 (low spin)<br>0.65 (high spin)
Co	+2	VI	0.65 (low spin)<br>0.75 (high spin)
Ni	+2	VI	0.69
Cu	+2	VI	0.73
Zn	+2	IV	0.60
Cd	+2	VI	0.95
**REE**			
Sc	+3	VIII	0.87
Y	+3	VIII	1.02
La	+3	VIII	1.16
Ce	+3	VIII	1.14
Pr	+3	VIII	1.13
Nd	+3	VIII	1.11
Sm	+3	VIII	1.08
Eu	+3	VIII	1.07
Gd	+3	VIII	1.05
Tb	+3	VIII	1.04
Dy	+3	VIII	1.03
Ho	+3	VIII	1.02
Er	+3	VIII	1.00
Yb	+3	VIII	0.99

Table S5. Concentrations of REE in raw MSWIA and oxalate product (10 mM oxalate) and corresponding enrichment factors.

REE	Concentration in MSWIA (ppm)	Concentration in oxalate product (ppm)	Enrichment factor
Sc	3.12 ± 0.25	14.35 ± 1.42	4.60 ± 0.34
Y	12.70 ± 0.73	43.64 ± 3.12	3.44 ± 0.23
La	24.58 ± 1.23	98.51 ± 6.57	4.01 ± 0.24
Ce	44.58 ± 3.48	114.27 ± 8.89	2.56 ± 0.13
Pr	65.59 ± 3.76	881.57 ± 21.45	13.44 ± 0.94
Nd	219.78 ± 14.53	2187.76 ± 45.87	9.95 ± 0.64
Sm	2.08 ± 0.34	30.64 ± 3.04	14.73 ± 1.03
Eu	0.72 ± 0.13	3.40 ± 0.45	4.72 ± 0.45
Gd	2.26 ± 0.24	26.69 ± 1.74	11.81 ± 0.91
Tb	0.05 ± 0.01	0.69 ± 0.08	13.81 ± 0.95
Dy	14.01 ± 0.88	132.06 ± 10.2	9.43 ± 0.66
Ho	0.04 ± 0.01	0.28 ± 0.03	7.06 ± 0.68
Er	0.84 ± 0.14	4.47 ± 0.47	5.32 ± 0.31
Yb	0.69 ± 0.12	6.72 ± 0.87	9.73 ± 0.82
Total	391.06 ± 22.57	3545.03 ± 95.26	9.07 ± 0.73

Table S6. Mineralogical characteristics of synthesized zeolites determined by XRD.

ICDD #	Mineral Name	Chemical Formula	Weight%
Zeolite-A			
00-042-0215	Sodalite (Zeolite family)	$Na_8(AlSiO_4)_6(OH)_2(H_2O)_2$	51.1
00-054-0628	Cancrinite (Zeolite family)	$Na_{7.6}(AlSiO_4)_6(NO_3)_{1.6}(H_2O)_2$	38.9
04-024-5452	Cancrinite (Zeolite family)	$K_{0.4}Na_{7.1}Ca_{0.1}(AlSiO_4)_6(CO_3)O_{24}(H_2O)_{1.5}$	9.0
01-070-0424	Cancrinite (Zeolite family)	$K_9Na_{15}(AlSiO_4)_{18}(OH)_2(SO_4)_2(H_2O)_{6.8}$	1.0
Zeolite-B			
01-089-6324	Analcime (Zeolite family)	$Na_{0.93}(AlSi_2O_6)(H_2O)$	96.2
04-011-6754	Analcime (Zeolite family)	$Na_8Al_8Si_{16}O_{48}(H_2O)_8$	3.8

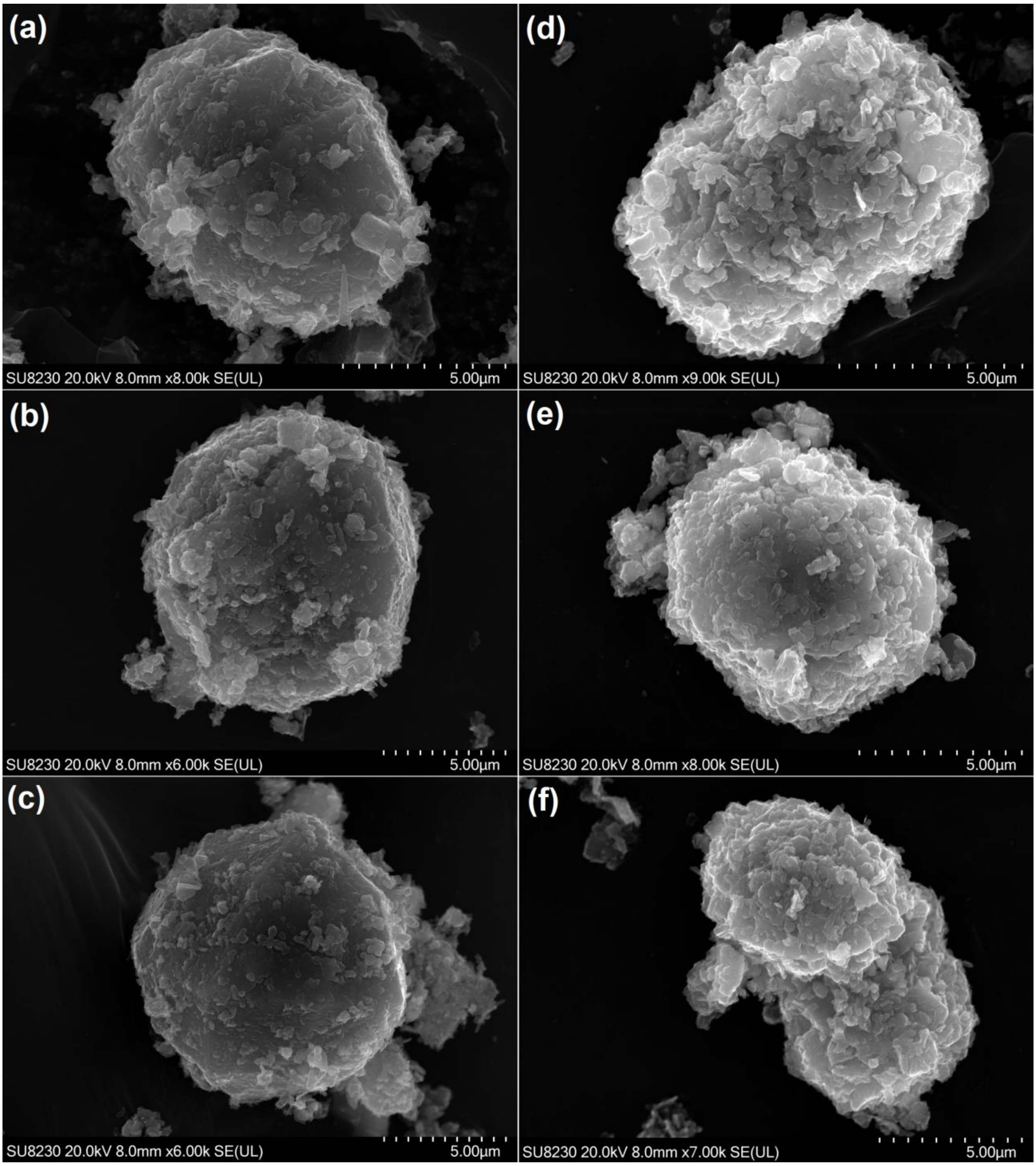

Figure S6. Additional SEM images of **(a-c)** zeolite-A and **(d-f)** zeolite-B showing different degrees of crystallization and particle sizes.

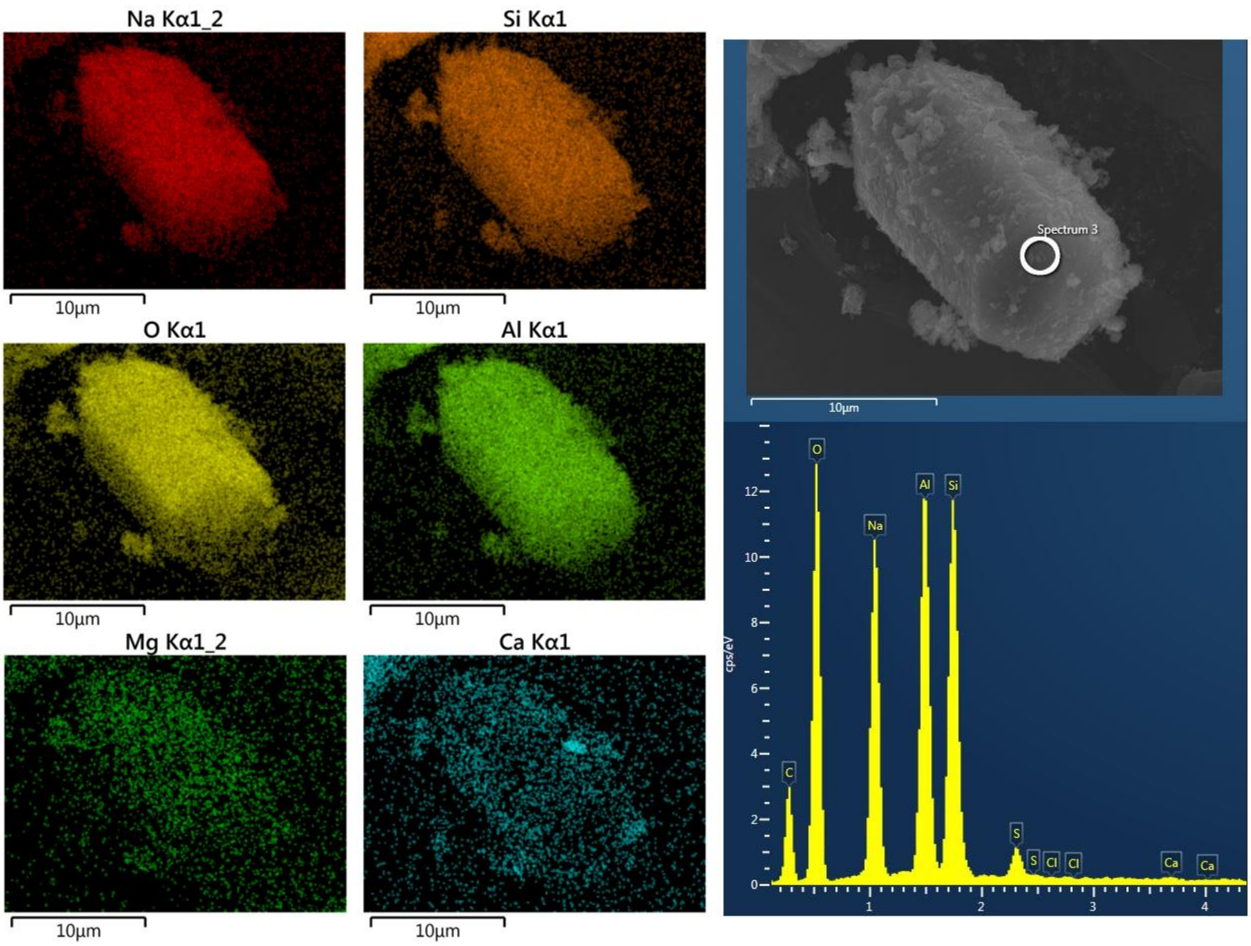

Figure S7. Elemental maps and point spectrum of the zeolite-A particle in **Figure 5c**.

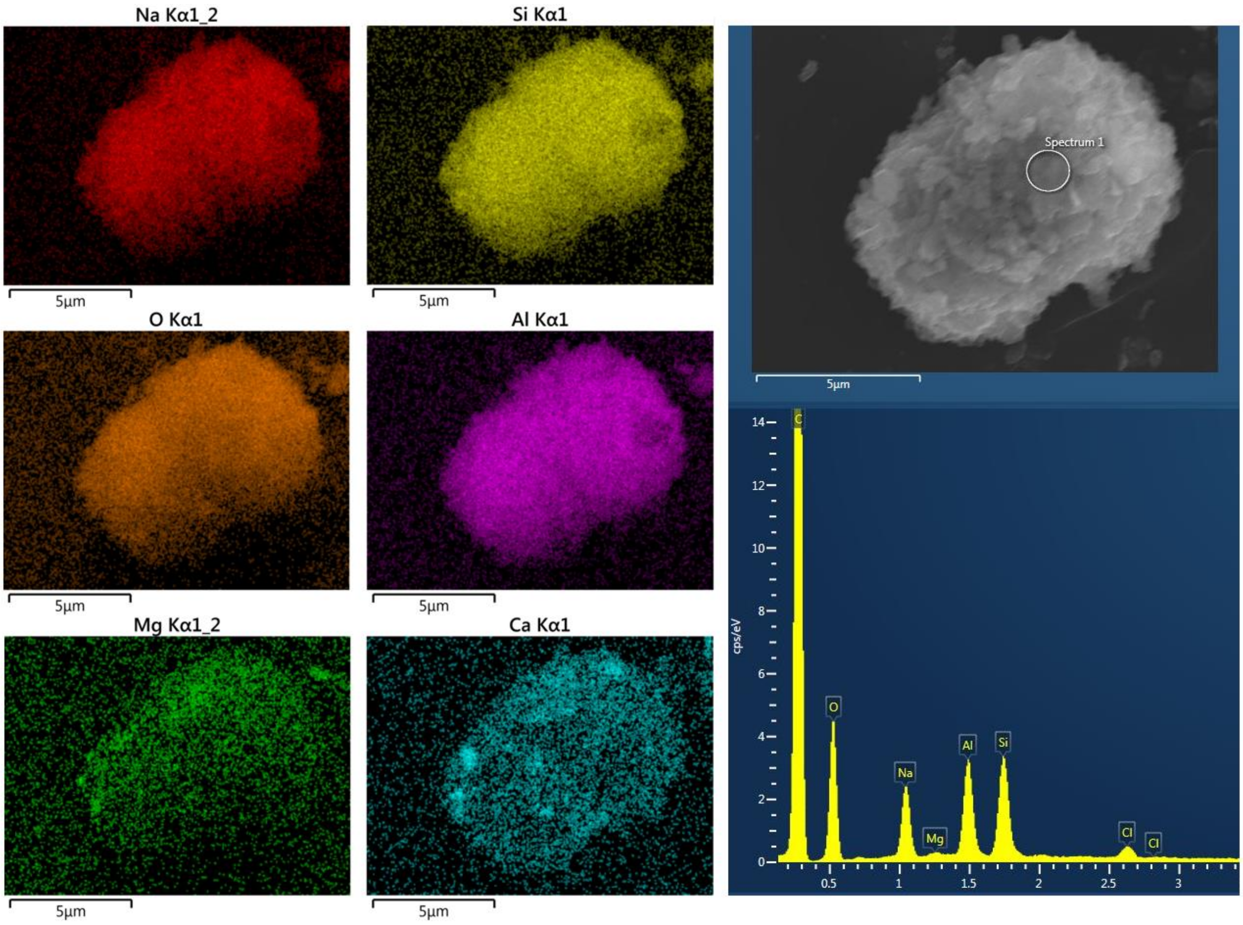

Figure S8. Elemental maps and point spectrum of the zeolite-B particle in **Figure S5d**.

Table S7. The concentrations of trace metals in the TCLP leachates (in comparison to EPA drinking water standards) and wastewaters of this three-step system with and without waste upcycling (unit: ppb).

Element	Wastewater			TCLP Leachate			EPA Standard
	Without upcycling (oxalate filtrate)	With upcycling (zeolite-A filtrate)	With upcycling (zeolite-B filtrate)	MSWIA	Zeolite-A	Zeolite-B	
Mg	14355.7±735.4	426.8±47.5	1506.5±85.3	9685.4±234.1	201.3±15.6	75.7±5.9	/
Al	53130.7±1263.5	14290.6±565.3	13768.9±510.3	24586.3±303.4	122.6±10.6	145.9±12.4	50-200**
Ca	134.1±14.4	12.6±1.4	12.0±1.3	48635.5±452.2	2.3±0.4	1.2±0.2	/
Cr	56.6±8.5	11.6±1.4	2.5±0.4	42.3±7.2	4.6±1.1	2.2±0.7	100*
Mn	1258.8±153.8	215.6±31.1	79.2±5.7	381.5±22.7	38.5±4.6	25.3±9.6	50**
Fe	19623.4±823.7	5640.7±156.9	4706.1±150.7	8336.9±136.4	258.4±15.3	315.9±26.8	300**
Co	109.3±8.6	11.0±0.9	3.0±0.4	31.5±2.5	3.3±0.3	2.8±0.2	/
Ni	120.3±12.5	19.5±3.1	12.6±1.0	53.2±4.2	1.2±0.3	3.3±0.2	/
Cu	5860.9±405.2	1758.5±113.2	1466.1±103.3	3736.8±112.4	125.3±11.6	133.1±11.8	1300*
Zn	36853.8±1932.7	11893.7±316.2	9566.0±324.6	7669.8±277.7	446.3±30.2	244.2±16.1	5000**
Cd	35.1±6.1	0.4±0.1	0.3±0.1	32.4±4.4	2.6±1.6	1.3±0.7	5*
Pb	580.1±50.3	9.5±2.6	6.3±1.1	263.8±31.3	28.6±4.3	10.3±2.1	15

* Maximum contaminant level (MCL) of the national primary drinking water standards by EPA.

** MCL of the secondary drinking water standards by EPA.

References

(1) Shannon, R. D. Revised Effective Ionic Radii and Systematic Studies of Interatomic Distances in Halides and Chalcogenides. *Acta Crystallographica Section A* **1976**, *32* (5). https://doi.org/10.1107/S0567739476001551.
